# Evolocumab administration prior to Coronary Artery Bypass Grafting in patients with multivessel coronary artery disease (EVOCABG): study protocol for a randomized controlled clinical trial

**DOI:** 10.1186/s13063-022-06398-3

**Published:** 2022-05-23

**Authors:** Hye Rim Na, O Sung Kwon, Joon Kyu Kang, Yong Han Kim, Ju Yong Lim

**Affiliations:** 1 Department of Cardiothoracic Surgery, The Catholic University of Korea Seoul St. Mary’s Hospital, Seoul, Republic of Korea; 2 Department of Cardiology, The Catholic University of Korea Eunpyeong St. Mary’s Hospital, Seoul, Republic of Korea; 3 Department of Cardiothoracic Surgery, The Catholic University of Korea Eunpyeong St. Mary’s Hospital, Seoul, Republic of Korea

**Keywords:** PCSK9 inhibitor, Evolocumab, Coronary artery bypass grafting, Ischemia-reperfusion injury, Coronary artery disease, Major adverse cardiovascular events

## Abstract

**Background:**

Despite advances in surgical and postoperative care, myocardial injury or infarction (MI) is still a common complication in patients undergoing coronary artery bypass surgery (CABG). Several studies that aimed to reduce postoperative myocardial injury, including those investigating statin loading, have been conducted but did not indicate any clear benefits. Evolocumab, a PCSK9 inhibitor, has been reported to lower lipids and prevent ischemic events in various medical conditions. However, the effect of evolocumab in cardiovascular surgery has not been evaluated. The objective of this trial is to evaluate the cardioprotective effects of evolocumab in elective CABG patients with multivessel coronary artery disease.

**Methods:**

EVOCABG is a prospective, randomized, open, controlled, multicenter, superiority, phase III clinical trial. Patients with multivessel coronary artery disease without initial cardiac enzyme elevation will be recruited (*n*=100). Participants will be randomly allocated into two groups: a test group (evolocumab (140mg) administration once within 72 h before CABG) and a control group (no administration). The primary outcome is the change in peak levels of serum cardiac marker (troponin-I) within 3 days of CABG surgery compared to the baseline. Secondary outcomes include post-operative clinical events including death, myocardial infarction, heart failure, stroke, and atrial fibrillation.

**Discussion:**

This trial is the first prospective randomized controlled trial to demonstrate the efficacy of evolocumab in reducing ischemic-reperfusion injury in patients undergoing CABG. This trial will provide the first high-quality evidence for preoperative use of evolocumab in mitigating or preventing ischemic-reperfusion-related myocardial injury during the surgery.

**Trial registration:**

Clinical Research Information Service (CRIS) of the Republic of Korea KCT0005577. Registered on 4 November 2020.

## Administrative information

Note: the numbers in curly brackets in this protocol refer to SPIRIT checklist item numbers. The order of the items has been modified to group similar items (see http://www.equator-network.org/reporting-guidelines/spirit-2013-statement-defining-standard-protocol-items-for-clinical-trials/).

**Table:** 

Title {1}	Evolocumab administration prior to coronary artery bypass surgery in patients with multivessel coronary artery disease (EVOCABG): study protocol for a randomized controlled clinical trial
Trial registration {2a and 2b}	Clinical Research Information Service (CRIS) of the Republic of Korea KCT0005577. Registered on 4 November 2020
Protocol version {3}	Version 1.2 10-19-2020
Funding {4}	Catholic Medical Center Research Foundation
Author details {5a} Author	Hye Rim Na: Department of Cardiothoracic Surgery, The Catholic University of Korea Seoul St. Mary’s Hospital, Seoul, Republic of Korea O Sung Kwon: Department of Cardiology, The Catholic University of Korea Eunpyeong St. Mary’s Hospital, Seoul, Republic of Korea Joon Kyu Kang: Department of Cardiothoracic Surgery, The Catholic University of Korea Eunpyeong St. Mary’s Hospital, Seoul, Republic of Korea Yong Han Kim: Department of Cardiothoracic Surgery, The Catholic University of Korea Eunpyeong St. Mary’s Hospital, Seoul, Republic of Korea Ju Yong Lim: Department of Cardiothoracic Surgery, The Catholic University of Korea Seoul St. Mary’s Hospital, Seoul, Republic of Korea
Name and contact information for the trial sponsor {5b}	Investigator-initiated clinical trial Ju Yong Lim (Principal investigator) Phone: +82(10) 9613 7882 Email: millalim92@gmail.com
Role of sponsor {5c}	This is an investigator-initiated clinical trial.

## Introduction

### Background and rationale {6a}

Ischemic and reperfusion myocardial injury is still inevitable and is reported to be as high as 5–15% in patients that have undergone coronary artery bypass grafting (CABG) despite substantial advancements in cardiopulmonary support devices and surgical techniques [1–3].

Intraoperative myocardial injury is followed by critical complications, leading to high morbidity, mortality, and hospital expenses [4]. The mechanisms underlying ischemic and reperfusion myocardial injury during surgery are complex. In addition to direct ischemia, other factors such as thrombosis, oxygen free radicals, inflammation, endothelial dysfunction, changes in cellular metabolism, and immune reactions can lead to myocardial stunning and microvascular dysfunction [5].

A variety of clinical trials have been conducted to reduce myocardial injury during coronary artery bypass surgery. However, statins are currently the only medication approved for clinical use [6–8], although this is still an issue of debate [9, 10]. Statin, a hydroxymethylglutaryl-coenzyme A (HMG-CoA) reductase inhibitor, not only clears LDL cholesterol in the body, but is also considered cardioprotective due to its anti-inflammatory, antioxidant, and immunomodulatory effects [11–13].

Recently, PCSK9 inhibitors, a novel medication, were developed, which significantly lowers LDL cholesterol by inhibiting proprotein convertase subtilisin–kexin type 9 (PCSK9), a mediator known to degrade LDL receptors [14, 15]. Multiple studies have already proved its cardioprotective effects, and it is recommended as a standardized treatment for cardiovascular disease [15–19].

In addition to reducing cholesterol levels, PCSK9 inhibitor’s pleiotropic effects have allowed diverse applications in a variety of clinical situations. Its anti-inflammatory effects are attributed to its distribution on arterial vascular walls, which promote inflammation and atherosclerosis [20]. Furthermore, animal studies have indicated that PCSK9 inhibitors exhibit potential immunoregulatory properties, whereas septic shock models were shown to benefit from PCSK9 inhibitor administration [21]. Furthermore, Palee et al. reported that prior to administration reduces ischemic and reperfusion damage of the myocardium, thus improving cardiac function [22].

Given its potential cardioprotective effects, it can be speculated that PCSK9 inhibitors would be beneficial for patients undergoing cardiovascular surgery. However, the efficacy of PCSK9 inhibitors in patients undergoing cardiovascular surgery has not been demonstrated. We present a protocol designed to investigate the efficacy of a PCSK9 inhibitor (evolocumab) in preventing ischemic and reperfusion myocardial injury in multivessel coronary disease patients undergoing elective CABG surgery.

### Objectives {7}

This prospective, randomized, open, phase III clinical trial will evaluate the efficacy and safety of evolocumab administration prior to CABG surgery. A total of 100 patients will be recruited for a follow-up study of 1 month. The primary objective will be to assess the efficacy of evolocumab in protecting the myocardium from postoperative ischemia and reperfusion injury. The secondary objective will be to evaluate evolocumab and its relationship with various postoperative complications such as mortality, myocardial infarction, heart failure, stroke, and atrial fibrillation. Safety endpoints will be assessed based on serious adverse events, vital signs, laboratory results, relation to concomitant drugs, and clinical manifestations (left ventricular function and length of stay).

### Additional consent provisions for the collection and use of participant data and biological specimens {26b}

There are no additional studies to be done.

### Trial design {8}

In this randomized, phase III superiority controlled clinical trial, patients will be randomly assigned in a 1:1 ratio to one of two groups: a test group (evolocumab (140 mg) administration once within 72 h before CABG) and a control group (no administration). Daily blood tests will be performed during hospitalization, including those assessing cardiac markers, while major postoperative complications will be evaluated for 1 month. Medications such as aspirin and P2Y12 inhibitors (clopidogrel, prasugrel, ticagrelor) and recommended dose (2018 ACC/AHA guidelines) of statins will be given unless contraindicated [23].

## Methods

### Study setting {9}

EVOCABG will be conducted in a multicenter, university hospital (Catholic University of Korea’s Seoul St. Mary’s Hospital and Eunpyeong St. Mary’s Hospital), in the Department of Cardiothoracic Surgery. Patients from the outpatient clinic and referrals from other departments will have to provide informed consent to participate voluntarily. All participants will be assessed for eligibility based on inclusion and exclusion criteria.

### Eligibility criteria {10}

#### Primary inclusion criteria

Patients must meet the following criteria for inclusion:

1. Age ≥19 and <85 years old
2. Patients with multivessel coronary artery disease (CAD) without baseline cardiac enzyme elevation, electively scheduled for CABG
  2-1) Grossly visible stenosis ≥ 70% of two or more coronary arteries (≥ 50% for left main artery and proximal left anterior descending artery) on coronary angiography, which is indicated for two or more vessel graft anastomoses.
  2-2) Cardiac biomarkers indicative of myocardial infarction, such as CK-MB, troponin-I should be within normal range.
3. Patients who voluntarily participate and provide written informed consent signed by themselves or by a legal representative
4. Since elderly patients aged 75 years or older are also included in this trial, safety is considered the top priority during the selection and conduction of this trial.

#### Primary exclusion criteria

Patients meeting one or more criteria listed below shall be excluded from this trial:

1. Previously received open heart surgery
2. Scheduled for additional valve surgery due to valvular disease
3. Scheduled for emergency CABG due to myocardial infarction
4. Previously treated with evolocumab or other PCSK9 inhibitors within 12 weeks before registration
5. Active liver disease or decreased liver function (> threefold of the normal upper limit of AST/ALT)
6. Severe kidney dysfunction (eGFR <20 mL/min/1.73 m^2^)
7. Major organ transplant recipients (e.g., lungs, liver, heart, bone marrow, kidney, etc.)
8. CK-MB ≥ fivefold of the normal upper limit
9. Active infection or hematologic, nephrological, metabolic, gastrointestinal, and endocrine disorders (determined by the investigator)
10. Life-expectancy ≤ 1 year due to malignancy or fatal disease
11. Women of childbearing age who refuse to use medically accepted contraception or are diagnosed as pregnant on the first day of visit (unless chemical or surgical contraception has been performed or are determined to be premenopausal)
12. Pregnant, breastfeeding patients or those who are planning a pregnancy
13. Patients with dementia and impaired cognition or those who lack comprehension and do not comply with instructions given during the trial
14. Patient who suffer from alcohol or drug addiction
15. Previous participation in another trial within 6 months before registration
16. Unsuitable for participation for any other reasons, as judged by the investigator

### Who will take informed consent? {26a}

Patients with multivessel CAD without initial elevation of cardiac markers scheduled for CABG will be assessed for eligibility according to the inclusion and exclusion criteria. Eligible participants will receive a thorough explanation of the purpose of the study and all relevant information will be provided by investigators prior to enrollment. After providing written informed consent, the demographic and smoking history of patients will be recorded in addition to the date of initialization, with other basic physical information.

### Intervention description {11a}

Evolocumab, a or PCSK9-inhibitor, is a monoclonal antibody that inhibits proprotein convertase subtilisin-kexin type 9 (PCSK9). PCSK9 is a protease expressed mostly in hepatocytes, which binds to LDL receptors, which are involved in clearing lipids and reducing serum lipid concentration. PCSK9 inhibitors inhibit LDL receptor degradation, thereby increasing lipid clearance. It is widely known that hyperlipidemia is a major risk factor for CAD, and evolocumab has emerged as a novel drug that can reduce LDL cholesterol by approximately 60%.

### Explanation for choice of comparators {6b}

To evaluate the efficacy of evolocumab, the test group will be subcutaneously administered 140 mg of the drug within 72 h prior to the operation, and the control group will receive no treatment. However, all postoperative patients will receive the same standard care as well as post-CABG medications such as aspirin 75–100 mg, P2Y12 inhibitor (e.g., clopidogrel, prasugrel, ticagrelor), unless a high bleeding tendency is noted. In addition, moderate to high doses of statins will be given, as recommended by the 2018 ACC/AHA guidelines, unless contraindicated [23].

### Criteria for discontinuing or modifying allocated interventions {11b}

Discontinuation of evolocumab may be considered in the following situations:

1. Patient wishes to stop the administration of evolocumab
2. Investigator decides to discontinue the trial due to serious adverse events
3. Any violation of selection or exclusion criteria or the trial protocol
4. Occurrence of a serious disease during the clinical trial
5. Patient receives a drug(s) without the investigator’s consent, which may possibly obscure the efficacy and safety of evolocumab
6. Patient fails to appear for follow-up checks

### Strategies to improve adherence to interventions {11c}

Patients will be fully informed about the trial safety and methods before the administration of evolocumab. Adherence will be high due to the simple one-time administration of the drug prior to surgery. No other interventions will be given.

### Relevant concomitant care permitted or prohibited during the trial {11d}

There are no specific restrictions of concomitant care during the trial.

### Provisions for post-trial care {30}

The investigator may compensate for any harm or injury possibly caused by evolocumab administration during the trial. Patients who meet the criteria of compensation regulations are covered by insurance, in accordance with the regulations of safety and pharmaceuticals.

### Outcomes {12}

The primary outcome is the peak change in serum cardiac marker (troponin-I) within 3 days of CABG surgery compared to the baseline. The secondary outcomes include postoperative clinical events including death, myocardial infarction, heart failure, stroke, and atrial fibrillation.

### Participant timeline {13}

Table 1 shows the participant timeline.

**Table 1:** Participant timeline

	Study period									
Visit description	Enrollment	Allocation	Intervention	Postoperation					Discharge	Close-out
Visit number	V1			V2	V3	V4	V5	V6	V7	V8
Timepoint	− 72h~0			0h±2h	6h±2h	24h±6h	48h±6h	72h±6h	7 days ± 3 days	30 days ± 5 days
**Enrollment:**										
Eligibility screen	X									
Informed consent	X									
Allocation		X								
**Intervention:**										
Evolocumab administration			X							
No medication			X							
**Assessment:**										
Demographics	X									
Medical history	X									
Vital signs, physical data^a^	X			X	X	X	X	X	X	X
Urine HCG	X									
ECG^b^	X			X		X	X	X		
Laboratory tests	X			X	X	X	X	X	X	
Concomitant drug	X			X	X	X	X	X	X	X
Outcome variables^c^	X			X	X	X	X	X	X	
Transthoracic echocardiography	X							X		
Clinical results^d^, safety evaluation			X	X	X	X	X	X	X	X

*HCG* human chorionic gonadotropin, *ECG* electrocardiogram

^a^Physical data include height and body weight

^b^ECG can be additionally performed at the discretion of the physicians

^c^Outcomes variables include primary outcomes such as cardiac enzymes

^d^Clinical results include data on death, recurrent myocardial infarction, heart failure, stroke, new-onset atrial fibrillation, and prolonged hospitalization

### Sample size {14}

The objective of this trial is to evaluate whether evolocumab effectively prevents post-CABG myocardial injury in patients with multivessel CAD. The peak Troponin-I level during the first three postoperative days compared to the initial level was evaluated as the primary outcome. Based on studies by Kaushik et al. on the efficacy of statin in preventing myocardial injury in CABG patients, the difference between peak Troponin-I versus initial level was 1.00±1.34 ng/mL in the statin loaded group and 2.25±2.59 ng/mL in the control group [6]. Therefore, with the power of 0.8 and significance level of 0.05, the change of Troponin-I at the time of peak compared to the base point can be calculated as 1.00±1.34ng/mL in the evolocumab group and 2.25±2.59ng/mL in the control group. According to the Power Analysis & Sample Size (PASS) 16 program, a minimum of 45 people per group are required for validation and a potential dropout rate of 10%, 50 people per group, and a total of 100 people are required for this clinical trial.

### Recruitment {15}

Patients will be recruited from outpatient clinics and referrals from other departments during admission from the Catholic University of Korea’s Seoul St. Mary’s Hospital and Eunpyeong St. Mary’s Hospital.

## Assignment of interventions: allocation

### Sequence generation {16a}

Subjects who agree to participate in this trial will sequentially receive subject identification codes in the order of their screening visit. Patients are randomly assigned to ensure scientific validity, maximize comparability between groups, and avoid bias. Those who meet the eligibility criteria will be divided into two groups, with the stratified block randomization method used due to multi-institutional sampling. An independent statistician will produce random allocation numbers using the Statistical Analysis System (SAS) ver. 9.4, Microsoft Windows (SAS Institute Inc., Cary, NC, USA), which will then be distributed to the subjects.

### Concealment mechanism {16b}

Allocation is concealed in a sealed envelope.

### Implementation {16c}

In this trial, the test group will receive a subcutaneous injection of 140 mg of evolocumab, 72 h prior to surgery. The control group will not receive any placebo or medication.

## Assignment of interventions: blinding

### Who will be blinded {17a}

Patients and investigator will not be blinded.

### Procedure for unblinding if needed {17b}

The trial is open and there is no unblinding procedure.

## Data collection and management

### Plans for assessment and collection of outcomes {18a}

Efficacy and safety evaluation outcomes of this clinical trial are assessed according to the following variables:

#### Primary efficacy evaluation variable

1. Maximum change in troponin-I between baseline and peak level during the first 3 days after coronary artery bypass grafting

#### Secondary efficacy evaluation variables

1. AUC (area under the ROC curve) of troponin-I for 3 days after administration of evolocumab
2. Troponin-I level change at each visit compared to baseline level
3. Maximum change of CK-MB during the first 3 days after coronary artery bypass grafting compared to baseline level
4. AUC of CK-MB (creatine kinase MB) for 3 days after administration of evolocumab
5. Change in CK-MB levels at each visit compared to baseline level
6. Change in BNP (brain natriuretic peptide) levels at each visit compared to baseline level
7. Change in CRP (C-reactive protein) level at each visit compared to baseline level
8. Change in LVEF (left ventricular ejection fraction) 3 days after the procedure compared to baseline level
9. Cumulative mortality during a 1-month period after surgery
10. Cumulative recurrence rate of myocardial infarction during the first month after the procedure
11. Cumulative incidence of stroke (hemorrhagic stroke, ischemic stroke, transient ischemic attacks) during the first month after the procedure
12. Cumulative incidence of new atrial fibrillation during the first month after the procedure
13. Cumulative incidence of coronary revascularization (PCI or CABG) during the first month after the procedure

#### Safety outcome variables

1. Adverse event: The investigator will check for adverse drug reactions during hospital rounds and educate patients to report any adverse events. Information includes the date of occurrence and disappearance, severity, treatment, and other suspected drugs.
2. Vital sign: For each visit, blood pressure and heart rate will be recorded.
3. Laboratory tests: Complete blood count (hemoglobin, hematocrit, white blood cell, and platelet count) and blood chemistry (glucose, blood urea nitrogen, creatinine, estimated glomerular filtration rate, total protein, albumin, bilirubin, aspartate aminotransferase, alanine aminotransferase, alkaline phosphatase, γ-gamma glutamyl transpeptidase (visit 1), sodium, potassium, chloride, total cholesterol, low-density lipoprotein, high-density lipoprotein, triglycerides, lipoprotein (a), and hemoglobin A1c (visit 1)).
4. Concomitant drugs: All drugs taken 4 weeks before the screening visit will be recorded. After each visit, name, daily dose, route, duration, and reason for administration for each drug were recorded in the electronic case report form (eCRF).
5. Clinical results: Data on all clinical outcomes (death, recurrent myocardial infarction, heart failure, stroke, new-onset atrial fibrillation, and prolonged hospitalization) will be collected and relevant source documents will be submitted. Prolonged hospitalization has been defined as a hospital stay of more than 10 days. If the patient is admitted to another hospital, the investigator will try to collect as much data as possible. Follow-up will be continued for 1 month during outpatient visits, or through phone calls.

### Plans to promote participant retention and complete follow-up {18b}

Patients will be fully informed about the trial objectives and safety issues. Patients are allowed to drop out at any time during the trial; however, completion will be encouraged. Follow-up will be continued for 30 days after surgery and will emphasize the importance of routine postoperative checkups regardless of the study, especially during outpatient follow-up.

### Data management {19}

Information will be entered into the electronic case report form (eCRF), and access is given only to relevant medical staff within the hospital. Data collected from each subject during the investigation were accurate and complete on the eCRF. Electronic medical records will be stored for at least 3 years after trial termination and publication of the final report. Details of record preservation follow local regulations.

### Confidentiality {27}

All records of trial subjects remain confidential, not disclosed to the public, and protected by law of confidentiality. They will be registered based on identification number and initials in the eCRF, and their names will not be recorded. If the subject’s name is recorded in any other document, it must be deleted before a copy is taken. All records of the clinical trial, including those saved on a computer, should be kept safe, in accordance with the national data protection law.

In addition, subjects are fully informed that all personal information is handled in a strictly confidential manner, in accordance with the Data Protection Law, even when trial results are published or reported.

### Plans for collection, laboratory evaluation, and storage of biological specimens for genetic or molecular analysis in trial/future use {33}

There are no biological specimens to be collected nor stored.

## Statistical methods

### Statistical methods for primary and secondary outcomes {20a}

Statistical methods for this clinical trial will be two-tailed tests with a significance level of 0.05. Continuous variables will be presented as descriptive statistics (average, standard deviation, median, minimum, and maximum). Categorical variables will be presented as descriptive statistics (frequency and percentage).

#### Primary efficacy evaluation

The maximum change in troponin-I between peak and baseline during the first 3 days will be presented as descriptive statistics and tested with an independent t-test or Wilcoxon’s rank-sum test.

#### Secondary efficacy evaluation

AUC (area under the ROC curve) of both troponin-I and CK-MB for 3 days and maximum change of CK-MB over 3 days compared to baseline level after evolocumab administration will be tested with independent t-test and Wilcoxon’s rank sum test. Changes in troponin-I levels at each visit compared to the initial level will be analyzed with repeated ANOVA.

Changes in CK-MB, brain natriuretic peptide (BNP), and CRP level compared to baseline levels will be analyzed with repeated ANOVA.

Changes in left ventricular ejection fraction at 3 days from baseline will be analyzed with an independent t-test and Wilcoxon’s rank-sum test. For comparison within the group, a paired t-test or Wilcoxon’s signed rank test will be used.

Cumulative mortality, recurrence rate of myocardial infarction, incidence of stroke, new atrial fibrillation, and incidence of coronary revascularization 1 month after the procedure will be analyzed using the Kaplan-Meier method. Differences between the two groups will be tested using the log-rank test. In this analysis, no missing values will be used.

### Interim analyses {21b}

There are no interim analyses.

### Methods for additional analyses (e.g., subgroup analyses) {20b}

There are no additional analyses.

### Methods in analysis to handle protocol non-adherence and any statistical methods to handle missing data {20c}

Sensitivity analysis will be performed to handle protocol non-adherence. Both “as treated” and “per protocol” analyses will be conducted excluding any protocol violation and deviation cases. If data is lost or a patient drops out during the trial, the most recent data will be analyzed as if it were obtained at that time (last service carried forward analysis). However, during survival analysis, missing data will not be replaced, and analysis will be performed using previously collected data.

### Plans to access the full protocol, participant-level data, and statistical code {31c}

Medical information of individual subjects obtained from this clinical study is confidential, and disclosing it to a third party other than those permitted access will be strictly prohibited. However, in certain circumstances, information may be provided to the individual's doctor or other appropriate medical personnel responsible for the well-being of the subject. In addition, data generated as a result of this trial will be disclosed to the IRB and the Ministry of Food and Drug Safety upon request for due diligence.

## Oversight and monitoring

### Composition of the coordinating center and trial steering committee {5d}

#### Executive Committee (EC)

The Executive Committee (EC) approves the final trial design, protocol, and participating organization. The results for the presentation, paper, and additional secondary projects will be reviewed. Members of the executive committee consist of the principal investigator of each institute and the organizer of the clinical trial.

#### Clinical Case Review Committee

The Clinical case review committee (CCRC) consists of interventional and non-interventional cardiologists. The committee is responsible for the clinical endpoint adjudication process and establishing definite protocol-based criteria for major clinical events and evaluation variables. Regular meetings will be held to discuss and evaluate all clinical trial events during the trial, while the primary results will be obscured to all members of this committee.

#### Data cooperation and management of trial institutions

The client and/or delegated authority will conduct data management and institutional management.

### Composition of the data monitoring committee, role, and reporting structure {21a}

Monitoring of participating institutions will be conducted by the Data Coordinating Center (DCC). Primary and secondary outcomes, adverse events, pre-specified key variables, and randomly selected 10% of subjects will be 100% monitored via correlation to their medical records.

Each institution will be routinely visited to ensure that the trial regulations and plans are adhered to. Contacting all potential institutions is essential to inform the potential investigators and related sub-investigators about the investigational drugs, medical devices, plans, regulations, expectations, subject registration, subject selection, informed consent, and required clinical data and records. These institutions are then evaluated for their capacity to provide enough subjects, a sub-investigator or manager, and sufficient support for data management. Appropriate training and education for sub-investigator(s) during initial visits were performed prior to the start of the trial. DCC monitors will ensure adherence to the initial plan, appropriate subject registration, accurate data reporting, and records of investigational drugs and medical devices. Finally, when the trial ends, the monitoring agent makes a last visit to collect all relevant documents, verify that they are complete, review storage requirements with the investigator, and ensure that all requirements are met.

The reporting structure is described in the following table. DCC may help with the establishment of the reporting format, but the final decision is made by the investigator.

**Table:** 

**Types of report**	**Reported subject**	**Reporting deadline**
Serious adverse event	IRB	In accordance with local regulations
	DCC	Death or life-threatening events: within 7 working days
		Others: within 15 working days
Interim report	IRB DCC IRB	In accordance with local regulations
Violation of the trial plan	IRB	In accordance with local regulations
	DCC	Within 7 working days
Final summary report	DCC	Within a month

IRB: Institutional Review Board; DCC: Data Coordinating Center

### Adverse event reporting and harms {22}

#### Adverse events

Injection-site reactions and allergic reactions may occur after a single subcutaneous injection of evolocumab, but are rare. It has been reported that myalgia, upper respiratory infection symptoms, alanine aminotransferase (ALT), and creatine kinase (CK) elevation may occur during long-term use.

#### Recording severe adverse events

At the final evaluation, duration, severity, association with evolocumab, initial management, final observational results, existence of other treatments, and severe adverse events will be recorded.

#### Severe adverse event reporting method

If any significant adverse event occurs, it will be reported as follows:

1. Who: The investigator
2. How: By fax or e-mail after filling out the serious adverse event (SAE) form (verbal report first before documented report if necessary)
3. What: Severe adverse event
4. When: Within 24 h of recognition by investigator
5. To whom: IRB of each institution

In cases of death or life-threatening adverse events, the investigator shall report within 7 days of recognition and additional detailed information within 8 days from initial reporting. In all other severe and unexpected adverse events, the investigator will fill out the SAE form and submit it to the IRB within 15 days of recognition. Follow-up evaluation should continue even when the subject does not recover from the adverse event at the end of the trial.

The investigator should complete the medical records regardless of its relevance to the clinical trial drug. The information provided should be sufficient for independent medical assessment. If necessary, the safety manager may contact the investigator for verification. As soon as additional data are collected, the investigator shall deliver it to the client, and all adverse events must be tracked until they are resolved or stabilized.

Hospitalization or surgery for pre-existing or underlying diseases is not considered an adverse event. However, if the subject’s baseline condition deteriorates during the trial, it can be considered an adverse event. All clinical events, including myocardial infarction, cerebral infarction, unplanned coronary revascularization, and death, will be centrally adjudicated, despite its contribution to the trial results. The investigator is responsible for providing all relevant evidence documents to the DCC so that the CCRC can independently assess each event. On a regular basis, the database will undergo quality control for cardiac enzymes and electrocardiograms, and the investigator shall submit all copies of these results to the CCRC.

### Frequency and plans for auditing trial conduct {23}

The auditing trial was completed on the 28th of July. Additional auditing was not planned.

### Plans for communicating important protocol amendments to relevant parties (e.g., trial participants and ethical committees) {25}

The investigator will obtain prior approval from the IRB and the Ministry of Food and Drug Safety for substantial amendments that interfere with the main procedures or plan of the clinical trial if it does not significantly affect administrative procedures or safety.

### Dissemination plans {31a}

No results will be published until the final analysis and evaluation is complete. Decisions regarding announcing the results will be confirmed by the executive committee (EC).

EC reviews the primary result and (1) decides whether to distribute information early at national and international science conferences, and (2) provides data to the author. The author first prepares an official presentation, then seeks advice from the EC, and finally submits the corresponding document to the EC. However, no test results need to be announced without EC approval.

## Discussion

Emerging basic studies have suggested the pleiotropic effects of PCSK9 inhibitors. With various mechanisms, PCSK9 inhibition prior to ischemia was proven to improve cardiac function and ischemia-reperfusion injury in animal models. Our protocol is to assess this cardioprotective effect of PCSK9 inhibitors in the clinical field, because ischemia-reperfusion injury is inevitable in CABG. Therefore, somewhat cardiac enzyme elevation related to this ischemia-reperfusion injury occurs in almost patients undergoing CABG within the 48h postoperative period. In addition, perioperative MI may occur due to grafts failure following CABG. Measurement of cardiac enzymes in the 48h postoperative period is recommended to monitor possible perioperative MI. Therefore, cardiac enzymes will be measured for 3 days after CABG to evaluate the overall ischemia-reperfusion injury developed by CABG. The potential cardioprotective benefit of PCSK9 inhibitors will be assessed by differences of enzyme elevation in test and control groups.

### Strengths

This trial is the first prospective randomized controlled trial to demonstrate the efficacy of evolocumab in reducing ischemia-reperfusion injury in patients undergoing coronary artery bypass grafting. This will be the first trial to provide high-quality evidence for the preoperative use of evolocumab to protect against ischemic reperfusion-related myocardial injury during surgery. It also suggests the potential benefit of preoperative use of evolocumab in the clinical outcomes following CABG.

### Limitations

This trial has several potential limitations, which may have biased the study results. First, as evolocumab must be administered subcutaneously with a unique type of syringe, blinding was not possible for both patients and investigators. Second, the peak therapeutic effect of evolocumab has been reported to occur approximately 7 days after administration. However, surgery is usually performed within a few days after patient referral because of the cost-effectiveness for both hospitals and patients. Therefore, the time frame of our study drug administration, 72 h prior to the surgery, will not be optimal to demonstrate the effect of the study drug.

## Trial status

Patient recruitment began on January 25, 2021. Currently (December 27), we have included 23 patients. We expect to complete the trial by around March 31, 2023.

## Data Availability

Data or materials during the study shall be available by the investigator or the corresponding author upon reasonable request.

## Abbreviations

ADL: Activities of daily living
AE: Adverse event
ALT: Alanine transaminase
AST: Aspartate transaminase
AUC: Area under the curve
BNP: Brain natriuretic peptide
BUN: Blood urea nitrogen
CABG: Coronary artery bypass grafting
CCRC: Clinical Case Review Committee
CK-MB: Creatine kinase MB isoenzyme
DCC: Data coordinating center
DCFs: Data Clarification Form
EC: Executive Committee
ECG: Electrocardiogram
eCRF: Electronic case report form
F/U: Follow-up
FAS: Full analysis set
GCP: Good Clinical Practice
HCG: Human chorionic gonadotropin
HDL: High-density lipoprotein cholesterol
hsCRP: High-sensitivity C-reactive protein
ICH: International Conference on Harmonization
IRB: Institutional Review Board
ITT: Intention to treat
IWRS: Intracive web-based response system
LDL: Low-density lipoprotein cholesterol
LVEF: Left ventricular ejection fraction
MFDS: Ministry of Food and Drug Safety
Op: Operation
PCI: Percutaneous coronary intervention
PCSK9: Proprotein convertase subtilisin-kexin type 9
PPS: Per protocol set
SAE: Serious adverse event
SOP: Standard operating procedure

## References

[1] Gummert JF, Funkat A, Beckmann A, Schiller W, Hekmat K, Ernst M (2009). Cardiac Surgery in Germany during 2008. A report on behalf of the german society for thoracic and cardiovascular surgery. Thorac Cardiovasc Surg.

[2] Kim LK, Looser P, Swaminathan RV, Minutello RM, Wong SC, Girardi L (2016). Outcomes in patients undergoing coronary artery bypass graft surgery in the United States based on hospital volume, 2007 to 2011. J Thorac Cardiovasc Surg.

[3] Serruys PW, Morice MC, Kappetein AP, Colombo A, Holmes DR, Mack MJ (2009). Percutaneous coronary intervention versus coronary-artery bypass grafting for severe coronary artery disease. N Engl J Med.

[4] LaPar DJ, Crosby IK, Rich JB, Fonner E, Kron IL, Ailawadi G (2013). A contemporary cost analysis of postoperative morbidity after coronary artery bypass grafting with and without concomitant aortic valve replacement to improve patient quality and cost-effective care. Ann Thorac Surg.

[5] Verma S, Fedak PW, Weisel RD, Butany J, Rao V, Maitland A (2002). Fundamentals of reperfusion injury for the clinical cardiologist. Circulation..

[6] Kaushik A, Kapoor A, Agarwal SK, Pande S, Tewari P, Majumdar G (2020). Statin reload before off-pump coronary artery bypass graft: Effect on biomarker release kinetics. Ann Card Anaesth.

[7] Mannacio VA, Iorio D, De Amicis V, Di Lello F, Musumeci F (2008). Effect of rosuvastatin pretreatment on myocardial damage after coronary surgery: a randomized trial. J Thorac Cardiovasc Surg.

[8] Pan W, Pintar T, Anton J, Lee VV, Vaughn WK, Collard CD (2004). Statins are associated with a reduced incidence of perioperative mortality after coronary artery bypass graft surgery. Circulation..

[9] Kuhn EW, Slottosch I, Wahlers T, Liakopoulos OJ. Preoperative statin therapy for patients undergoing cardiac surgery. Cochrane Database Syst Rev. 2015;(8):CD008493. 10.1002/14651858.CD008493.pub3 26270008

[10] Zheng Z, Jayaram R, Jiang L, Emberson J, Zhao Y, Li Q (2016). Perioperative Rosuvastatin in Cardiac Surgery. N Engl J Med.

[11] Albert MA, Danielson E, Rifai N, Ridker PM, Investigators P (2001). Effect of statin therapy on C-reactive protein levels: the pravastatin inflammation/CRP evaluation (PRINCE): a randomized trial and cohort study. JAMA..

[12] Brull DJ, Sanders J, Rumley A, Lowe GD, Humphries SE, Montgomery HE (2001). Statin therapy and the acute inflammatory response after coronary artery bypass grafting. Am J Cardiol.

[13] Schwartz GG, Steg PG, Szarek M, Bhatt DL, Bittner VA, Diaz R (2018). Alirocumab and Cardiovascular Outcomes after Acute Coronary Syndrome. N Engl J Med.

[14] Stein EA, Mellis S, Yancopoulos GD, Stahl N, Logan D, Smith WB (2012). Effect of a monoclonal antibody to PCSK9 on LDL cholesterol. N Engl J Med.

[15] Navarese EP, Kolodziejczak M, Kereiakes DJ, Tantry US, O'Connor C, Gurbel PA (2016). Proprotein convertase subtilisin/kexin type 9 monoclonal antibodies for acute coronary syndrome: a narrative review. Ann Intern Med.

[16] Bittner V (2016). Pleiotropic Effects of PCSK9 (Proprotein Convertase Subtilisin/Kexin Type 9) Inhibitors?. Circulation..

[17] Robinson JG, Farnier M, Krempf M, Bergeron J, Luc G, Averna M (2015). Efficacy and safety of alirocumab in reducing lipids and cardiovascular events. N Engl J Med.

[18] Sabatine MS, Giugliano RP, Keech AC, Honarpour N, Wiviott SD, Murphy SA (2017). Evolocumab and Clinical Outcomes in Patients with Cardiovascular Disease. N Engl J Med.

[19] Sabatine MS, Giugliano RP, Wiviott SD, Raal FJ, Blom DJ, Robinson J (2015). Efficacy and safety of evolocumab in reducing lipids and cardiovascular events. N Engl J Med.

[20] Tang Z, Jiang L, Peng J, Ren Z, Wei D, Wu C (2012). PCSK9 siRNA suppresses the inflammatory response induced by oxLDL through inhibition of NF-kappaB activation in THP-1-derived macrophages. Int J Mol Med.

[21] Walley KR, Thain KR, Russell JA, Reilly MP, Meyer NJ, Ferguson JF (2014). PCSK9 is a critical regulator of the innate immune response and septic shock outcome. Sci Transl Med.

[22] Palee S, McSweeney CM, Maneechote C, Moisescu DM, Jaiwongkam T, Kerdphoo S (2019). PCSK9 inhibitor improves cardiac function and reduces infarct size in rats with ischaemia/reperfusion injury: benefits beyond lipid-lowering effects. J Cell Mol Med.

[23] Grundy SM, Stone NJ, Bailey AL, Beam C, Birtcher KK, Blumenthal RS (2019). 2018 AHA/ACC/AACVPR/AAPA/ABC/ACPM/ADA/AGS/APhA/ASPC/NLA/PCNA Guideline on the management of blood cholesterol: a Report of the American College of Cardiology/American Heart Association Task Force on Clinical Practice Guidelines. Circulation..

